# The Influence of Knowledge Base on the Dual-Innovation Performance of Firms

**DOI:** 10.3389/fpsyg.2022.879640

**Published:** 2022-05-27

**Authors:** Liping Zhang, Hailin Li, Chunpei Lin, Xiaoji Wan

**Affiliations:** ^1^College of Business Administration, Huaqiao University, Quanzhou, China; ^2^Development and Planning Department, Huaqiao University, Quanzhou, China

**Keywords:** dual-innovation performance, knowledge base, k-means clustering, CART algorithm, decision rules

## Abstract

Dual innovation, which includes exploratory innovation and exploitative innovation, is crucial for firms to obtain a sustainable competitive advantage. The knowledge base of firms greatly influences or even determines the scope, direction, and path of their dual-innovation activities, which drive their innovation process and produce different innovation performances. This study uses data source patents obtained by 285 focal firms in the Chinese new-energy vehicle industry in the period 2015–2020. Five knowledge-base features are selected by analyzing the correlation and multicollinearity, and four different firm clusters are found by using the k-means clustering algorithm. Based on the classification and regression tree (CART) algorithm, we mine the potential decision rules governing the dual-innovation performance of firms. The results show that the exploratory innovation performance of firms in different clusters is mainly affected by two different knowledge-base features. Knowledge-base scale is a key factor affecting the exploitative innovation performance of firms. Firms in different clusters can improve their dual-innovation performance by rationally tuning the combination of knowledge-base features.

## 1. Introduction

With the continuous advancement of global economic integration, the industrial economy is gradually moving toward a knowledge economy. Knowledge is becoming not only a critical production factor in scientific innovation but also an important strategic resource for firms. To obtain a sustainable competitive advantage in the market, firms not only need to acquire and absorb a significant amount of heterogeneous knowledge from outside the firm but also transform and upgrade their existing internal knowledge.

At present, many researchers divide knowledge into different categories. For example, Blackler (1995) divided knowledge into employee knowledge, knowledge embedded in experience and culture, coding knowledge, and organizational operation knowledge according to the object of knowledge storage. Nonaka and Takeuchi (1995) split knowledge into explicit knowledge and tacit knowledge according to the coding degree of knowledge. The former refers to knowledge that can be explained and understood by any individual with a relevant technical knowledge base both inside and outside the firm. However, the latter reflects the knowledge that is implicit in action and closely related to the specific environment. Tacit knowledge can be converted into explicit knowledge through coding, but explicit knowledge cannot completely replace all content included in tacit knowledge (Robin and Dominique, 1997). Toward this end, Kogut and Zander (1992) proposed a definition of a knowledge base. They believed that various information and skills in a knowledge base should be able to contribute significantly to maintaining the survival and development of firms. In fact, the knowledge base has characteristics of persistence, complexity, and transferability. Meanwhile, it is also difficult to be cracked, imitated, and chased by other competitors. Therefore, the knowledge base is not only a decisive resource for firms but also an important component of their sustainable competitive advantage (Yu and Yan, 2021; Low and Ho, 2016). A firm with a stronger knowledge base can predict the future more accurately. In addition, it can also develop new opportunities and further discover potential business value in the environment. In a word, the knowledge base of a firm plays an important role in the diversification and specialization of its innovation activities, which is one of the most important sources of competitive advantage and is a major factor in determining innovation performance.

In reality, there are many forms of innovation. Firms carrying out relevant innovation activities may obtain sustainable competitive advantages (Pomegbe et al., 2020). As an important innovation form, dual innovation can be divided into exploratory innovation and exploitative innovation based on the knowledge source used in the process of innovation. Exploratory innovation refers to the process whereby firms break through their own boundaries and acquire new knowledge from the external environment. Exploitative innovation, in contrast, is the process whereby firms deeply understand and integrate the mining of the existing knowledge stock (Zhang and Luo, 2020; Yang et al., 2021). In the process of exploratory innovation, although firms may face higher costs and risks, they can venture into more technical fields, which can significantly accelerate their economic growth. However, when firms implement exploitative innovation, due to the lower cost and risk of R&D, not only can they enhance their technical capabilities and competitive advantages in their current field but they can also increase their profits (Garcia-Vega, 2006; Dobrzanski et al., 2021). Dual innovation is thus extremely important for promoting the long-term survival and development of firms.

In the process of a firm's technological innovation, its knowledge base plays an important role (Su et al., 2021; Wang et al., 2022). Both the appearance of new knowledge and the application of existing knowledge are sources of a firm's technological innovation (Grant, 1996). Given that exploratory innovation requires a firm to obtain diverse knowledge from the outside, and exploitative innovation emphasizes the innovation, integration, and improvement of existing knowledge and technology (Yang et al., 2021), the dual innovation of a firm is related to its knowledge base. Knowledge-base features affect the opportunity and potential of the combination of knowledge elements of a firm and also affect its dual-innovation performance. Brusoni and Geuna (2003) revealed the important role played by the breadth and depth of a firm's knowledge base for its technological innovation activities and pointed out that the knowledge base is an important source of heterogeneity in innovation activities. Saviotti (2005) studied the relationship between knowledge-base scale and knowledge-base consistency in US pharmaceutical firms in the 1990s and their corresponding innovation performance. His study results show that both knowledge-base scale and knowledge-base consistency improve the innovation performance of firms. Further differentiating the impact of knowledge-base features on the performance of different types of innovations, Dibiaggio et al. (2014) reported that knowledge complementarity correlates strongly with the innovation performance of firms, whereas knowledge substitutability negatively affects a firm's innovation performance. In particular, high-level knowledge substitutability improves the exploratory innovation performance of firms. Carnabuci and Operti (2013) focused on the relationship between the knowledge reorganization capability of firms and the exploitative innovation performance. They report that knowledge diversification enhances the capacity of firms to reorganize their knowledge and further improves their performance in exploitative innovation.

To summarize, research shows that the importance of a firm's knowledge base to the dual-innovation activities are now regarded as an indisputable fact. However, relatively few studies have analyzed how a firm's knowledge base affects its dual-innovation performance. In addition, the mechanism whereby a firm's knowledge base internally influences its dual-innovation performance needs to be further investigated. Considering the fact that massive data contains a large amount of valuable information and knowledge, similar to the application of big data in the field of the Internet of Things (Kovacova and Lewis, 2021; Durana et al., 2021), Artificial Intelligence (Kovacova et al., 2020; Lazaroiu et al., 2021), and Intelligent Process Planning (Kovacova and Lazaroiu, 2021; Valaskova et al., 2021), this study will solve the following problems through the data-driven analysis method.

1. How does one scientifically select the features of a firm's knowledge base from various variables?
2. Based on selected knowledge-base features, how does one divide firms into different clusters?
3. For firms with different knowledge-base features, how is the original knowledge-base structure inherently related to and influenced by the firm's dual-innovation performance? In addition, what types of knowledge-base features are more conducive to improving the firm's dual-innovation performance?

This study makes the following main contributions:

1. Knowledge-base features affecting the dual-innovation performance of firms are objectively selected, and, based on these, firms are divided into four different clusters by using the k-means clustering algorithm. The detailed differences among them are discussed.
2. Decision trees for the exploratory and exploitative innovation performance in different clusters are constructed by using the classification and regression tree (CART) algorithm.
3. The complex mechanism whereby a firm's knowledge base inherently influences its dual-innovation performance is analyzed for different clusters of firms, and decision rules that affect a firm's dual-innovation performance is proposed.

The remainder of this study is organized as follows. Section 2 introduces the k-means clustering and CART algorithms. Section 3 describes how the sample data are cleaned and selects and measures related variables. Section 4 designs the research framework and introduces the processes involved in this study. Decision rules for the various clusters of firms are presented in Section 5, and, finally, we conclude the paper by discussing the research results in Section 6.

## 2. Preliminaries

This section introduces the k-means clustering algorithm and the CART algorithm, which are the main methods used in this research.

### 2.1. k-means Clustering Algorithm

The k-means clustering algorithm is a traditional classical clustering algorithm in the field of data mining (Mengyao, 2020; Li, 2021; Li and Liu, 2021) that can divide the sample dataset into several disjoint clusters. The members of a given cluster are similar but differ from those in other clusters. The principle of the k-means clustering algorithm is relatively simple and is easy to satisfy. The basic steps of this algorithm are as follows: first, we use the elbow algorithm (Mouton et al., 2020) to determine the optimal clustering number k. Next, we randomly select k sample data from the dataset as the center of the initial cluster and then calculate the distance between all sample data and the centers of each cluster. When a sample datum is close to the center of a given cluster, we put it into the cluster. Finally, for the newly created clusters, we regard the mean of the sample data in each cluster as the center of the new cluster. The center of each cluster is continuously updated, and the algorithm terminates once the allocation results of all sample data no longer change. Given that k-means has a simple principle and a strong interpretation, we use it herein to sort firms into different clusters.

### 2.2. CART Algorithm

The decision tree is a popular means of machine learning that is easy to understand and explain. It can not only be used for classification but also for regression. Common decision tree algorithms include ID3 (Quinlan, 1986; Zhai et al., 2018), C4.5 (Quinlan, 1995; Lo et al., 2019), and CART (Breiman et al., 1984; Garcła et al., 2019; Zhao et al., 2020). They all follow the top-down approach and construct the decision tree from sets of training tuples and their associated class labels. Given that ID3 and C4.5 may generate numerous branches, they produce decision rules that are difficult to explain. Compared with ID3 and C4.5, the CART algorithm uses the Gini coefficient to select and divide attributes and uses binary recursive segmentation technology to generate a concise structure that generates understandable rules with less cost. Therefore, the CART algorithm is applied herein to reveal the multi-factor cross-effects of the dual-innovation performance of firms.

The classification and regression tree algorithm involves the processes of splitting, pruning, and tree selection. Splitting is a type of binary recursive process that features input and prediction, which can be either continuous or discrete. In addition, it continues to grow without stopping rules. The pruning process uses cost-complexity pruning: it starts from the largest tree and chooses the split node that, as the next pruning object, contributes the least to the overall performance until only the root node remains. Given that the CART algorithm may generate a series of nested pruning trees, an optimal decision tree must be selected. The process of selecting such a tree generally uses a separate test set to evaluate the predictive performance of each pruned tree and selects the optimal decision tree by performing cross-validation of the pruned subtrees. In short, the CART algorithm has two main steps: decision-tree generation and decision-tree pruning. The former generates the largest possible decision tree based on the training set, and the latter prunes the generated tree and selects the optimal subtree based on the test set. The minimum-loss function serves as the pruning criterion. For the detailed steps of this algorithm, please refer to the literature (Breiman et al., 1984).

## 3. Data and Variables

To resolve the questions put forward proposed in this study, we must first introduce some sample data and related variables.

### 3.1. Data Sourcing and Processing

#### 3.1.1. Data Sourcing

As a type of knowledge asset of firms, patents have huge commercial value and are thus an important means to enhance a firm's competitive advantage. In addition, given that the number of patents held by an organization (firms, regions, or even countries) can reflect its capacity to innovate, patents can be viewed as an important metric of innovation output and of the overall performance of firms. This study thus uses as sample data patent data from the field of Chinese new-energy vehicles. This choice is justified by the fact that the new-energy-vehicle industry is an emerging industry with high technological content with a fast technological iteration cycle and relatively intensive innovation activities. These factors facilitate research on the innovation performance of firms. Next, to determine who owns inventions and creations, firms in this industry usually go through legal procedures to apply to the patent office for patent authorization to ensure that their intellectual property rights on new technologies and achievements are protected by the national legal framework. The patent data in this industry thus well embodies the relationship between the knowledge base and the dual-innovation performance of Chinese firms. In addition, the vigorous development of the new-energy vehicle is a significant strategic initiative in numerous countries. For example, the “Development Plan for the New Energy Vehicle Industry (2021–2035)” was issued by the General Office of the State Council of China on 2 November 2020 to promote the high-quality and sustainable development of the Chinese new-energy-vehicle industry. The issue on how to improve the capacity of technological innovation and the performance of firms in the field of new-energy vehicles has thus become an important focus in both academic and industrial circles.

The sample patent data used herein mainly come from the global patent database PatSnap, which contains patent data from 126 countries and regions around the world from 1,790 to the present. At present, 160 million patents and 140 million copyrights can be searched in this database. The database is updated in a timely manner and contains a significant amount of global patent data, which is extremely convenient for understanding and studying domestic and foreign technologies and global patterns. In previous studies, some scholars used pending patents as sample data. However, because some pending patents may not be granted and the time span from patent application to authorization ranges from a few months to nearly 48 months, we use sample data as only authorized patents with high technical content. In addition, because industrial design patents do not have an International Patent Classification (IPC), the relationship between knowledge-base features and the dual-innovation performance of firms may be misleading. In addition, compared with invention patents and utility model patents, the technical content of industrial design patents are lower and they are fewer in number. Thus, we use only invention patents and utility model patents in this research.

#### 3.1.2. Data Processing

Given that knowledge base affects a firm's dual-innovation performance only for a certain time period (March, 1991), we use patents from year *t*−5 to year *t*−3 to calculate the knowledge-base features of firms and use patents from year *t*−2 to year *t* to measure the exploratory and exploitative innovation performance of firms. To ensure the timeliness and validity of the original patent data and reduce the impact of noise caused by changes in the technical environment, we select as sample data invention and utility model patents in the field of new-energy vehicles that were granted from 1 January 2015 to 31 December 2020. The patents granted in the first 3 years are used to obtain knowledge-base features of firms, and the patents granted in the last 3 years are used to measure the dual-innovation performance of firms.

After searching a series of topics related to new-energy vehicles, a total of 210,540 patents were retrieved from PatSnap, including 195,535 independent patents and 15,505 cooperative patents. Furthermore, 62,417 patents were granted from 1 January 2015 to 31 December 2017, and 148,123 patents were granted from 1 January 2018 to 31 December 2020. To obtain focal firms, we first select firms that obtained five or more cooperative patents in the first 3 years and then match them with firms from the last 3 years. Finally, we choose 285 focal firms as the research object of this study.

To ensure the reliability and accuracy of patents, we cleaned the sample data. For example, the patent holders “State Grid Co., Ltd.” is the same as the “State Grid Corporation,” and the “China National Petroleum Co., Ltd.,” is the same as the “China National Petroleum Corporation.” Therefore, their data should be merged. In addition, due to differences in symbol format, some patent holders such as “Robert•Bosch Co., Ltd.” and “Robert-Bosch Co., Ltd.” are often viewed as two different patentees. In fact, data from firms with the same name or highly similar names should also be merged. In the process of cleaning the sample data, we used platforms such as “Aiqicha.baidu.com” to eliminate dualities caused by patentee names. After data cleaning and name matching, we are left with 24,311 patents granted from 1 January 2015 to 31 December 2020 to 285 focal firms.

### 3.2. Selection and Measurement of Knowledge-Base Features

A firm's knowledge base is the most unique and important resource the firm has for implementing innovation activities. It plays an important role in promoting the diversification and specialization of a firm's innovation activities. Firms with a stronger knowledge base can discover and develop new business opportunities in a timely manner, which improves their innovation performance. However, the rigidity of a firm's cognitive model and the increase in knowledge-transaction and management costs may create significant uncertainty for a firm, which is not conducive to improving the firm's innovation performance. In terms of these complex and uncertain characteristics, many researchers study how, from a resource-based view, a knowledge-based view, and absorptive capacity, a firm's knowledge base affects its innovation performance.

Although significant research results have been found, the current research lacks a unified standard for the categorization of a firm's knowledge base. Most studies suffer from a certain degree of subjectivity and randomness in their categorization. Some researchers divide knowledge bases into knowledge-base breadth (KBB) and knowledge-base depth (KBD) according to the scope of knowledge coverage and familiarity with knowledge; in other words, the characteristics of knowledge development in the horizontal and vertical directions. They also studied how these two knowledge-base features affect a firm's innovation performance. For example, Wei et al. (2021) clarified the impact of KBB and KBD on digital innovation and examined how the relationships between IT capability and knowledge base are moderated by the institutional environments in which the firm operates. Mannucci and Yong (2018) found that KBD enhances the ability of firms to reconfigure similar knowledge and obtain unique output results in this field, which helps to improve the innovation performance of firms. KBB is also conducive to increasing the innovation performance of firms because it encourages firms to integrate diverse ideas into novel combinations. Yang et al. (2017) found that a firm's deep knowledge of a specific industry is imperative to the success of new products. The effect of KBB is contingent on KBD. In particular, a firm's deep knowledge in a specific field can systematically shift the KBB from having a negative effect to having a positive effect. Du (2021) found that a firm with a broad knowledge base is better able to develop incremental innovations matched with internal knowledge heterogeneity (KH) rather than external KH. Firms with high KBD benefit more from external KH than internal KH for fostering incremental innovations.

Given that an organization that innovates technologically is affected by the original technical knowledge base, existing research has confirmed that the organizational knowledge stock or the knowledge-base scale (KBS) is positively related to its technological innovation. Given that KBB reflects the degree of knowledge diversification and coverage (Zhou and Caroline Binxin, 2012) and that KBD displays the depth and complexity of the industry knowledge possessed by firms (Mannucci and Yong, 2018), KBS describes the degree of knowledge accumulation; that is, it embodies to a certain extent the overall characteristics of the knowledge base. Toward this end, the present study uses KBB, KBD, and KBS as knowledge-base features.

In addition, the diversity of the knowledge base, which reflects the distribution and differentiation of knowledge resources, is also related to the firm's innovation performance. For example, Tang et al. (2021) pointed that the relationship between knowledge diversification and firm innovation is positive. The degree centralization of industrial knowledge networks, together with the coherence of firms' knowledge base, strengthens their positive relationship. Lin et al. (2006) found that a positive impact exists between the diversity of a firm's technological knowledge base and its innovation performance. They report that an improved diversity of a firm's technical knowledge base can reduce the average R&D cost, widen the scope of the technical resources mastered by the firm, and improve the firm's ability to identify, absorb, and apply new knowledge. However, some scholars argue that further improving the diversity of a firm's technical knowledge base will create more possibilities for combining knowledge elements. Thus, the complexity of firms' technological innovation activities will increase, as will their financial and material capabilities in innovation activities.

Meanwhile, firms' technological-innovation performance will be reduced further (Leten et al., 2007; Chen and Chang, 2012). Previous studies did not deeply explore the different categories of firms' technological knowledge base and the effects of its diversity. To reveal how different types of diversity affect the technological-innovation performance of firms, Krafft et al. (2011) divided the diversity of firms' technical knowledge base into knowledge-based unrelated diversity (KBUD) and knowledge-based related diversity (KBRD) according to the different resources allocated by firms in related or nonrelated technical fields.

Subsequently, scholars analyzed the relationship between KBUD, KBRD, and firms' innovation performance based on patent data. Based on archival data on 158 Chinese automobile companies from 1996 to 2010, Wen et al. (2021) reported that diversified unrelated knowledge enhances a firm's exploratory innovation outcomes, and the inter-firm R&D network that relies on diversity-related knowledge helps companies engage in exploitative innovation. Jungho et al. (2016) applied the unique panel data set of Korean manufacturing firms to analyze the relationship between technological diversification and firm growth and the conditioning role played in the relationship by firm-specific core-technology competence. They report an inverted-U relationship regardless of the type of technological diversification. However, for unrelated technological diversification, the inverted-U relationship weakens substantially for firms with high core-technology competence.

To summarize, the features such as breadth, depth, scale, and related and unrelated diversity of knowledge base affect firms' innovation performance. To facilitate further analysis, we define and measure them as follows.

#### 3.2.1. Knowledge-Base Breadth

Knowledge-base breadth (KBB) reflects the horizontal dimension of knowledge and measures the technical scope covered by the knowledge units of firms. At present, many different measurement methods are provided. To measure KBB, Zhou and Caroline Binxin (2012) used a maturity scale containing three items that focus on customer group, market knowledge, and diversity of R&D knowledge. Zhang and Baden-Fuller (2010) viewed each patent subcategory as separate technical fields and measured KBB by using the number of technical fields covered by patents in the past 3 years. Given the technical characteristics of the patent data used herein, we use the method Zhang and Baden-Fuller (2010) to measure KBB.

Assuming that the number of the first four IPC classification numbers (namely, IPC subclass) of patents granted to firm *i* in a year is *I*_*i*,year_, then the KBB of firm *i* is


(1) $$\begin{array}{l} {\text{KBB}}_{i}=\overset{2017}{\underset{\text{year}=2015}{\sum }}I_{i,\text{year}}=n_{i}, \end{array}$$

where *n*_*i*_ is the total number of the first four IPC classification numbers in patents granted to firm *i* in the first 3 years.

#### 3.2.2. Knowledge-Base Depth

Knowledge-base depth (KBD) embodies the familiarity of firms with existing technical knowledge and measures the vertical dimension of a firm's knowledge. In the current research, many measurement methods have been put forward. For example, Zhou and Caroline Binxin (2012) applied a maturity scale with four items to measure KBD that focuses on familiarity with the industry and internal knowledge. Lin and Wu (2010) used the dominant technical advantage and a variation coefficient to measure KBD. As done by Lin and Wu (2010) and Cantwell and Piscitello (2000), the present study measures KBD by applying the following steps:

1. Calculate the ratio of the number of authorized patents granted to firm *i* in technical field *j* (IPC subclass) to the total number of patents granted as follows:
  (2) $$\begin{array}{l} {\text{pro}}_{ij}=N_{ij}/\sum_{j=1}^{n_{i}}N_{ij}, \end{array}$$
  where *N*_*ij*_ is the number of patents granted to firm *i* in technical field *j* in the first 3 years, and *n*_*i*_ is the total number in IPC subclass of patents granted to firm *i* in all technical fields in the first 3 years.
2. The KBD of firm *i* is
  (3) $$\begin{array}{l} {\text{KBD}}_{i}=\sigma_{i}/\mu_{i}, \end{array}$$
  where μ_*i*_ and σ_*i*_ refer to the mean value and SD, respectively, of the ratio pro_*ij*_ for all technical fields.

#### 3.2.3. Knowledge-Base Scale

The knowledge-base scale (KBS) reflects the knowledge accumulation of firms, which measures the knowledge stock. This study uses the number of patents granted to a firm in the first 3 years as a measurement of their KBS. The formula to calculate the KBS of firm *i* is


(4) $$\begin{array}{l} {\text{KBS}}_{i}=\overset{n_{i}}{\underset{j=1}{\sum }}N_{ij}=N_{i}, \end{array}$$

where the definition of *N*_*ij*_ and *n*_*i*_ are the same as Equation (2).

#### 3.2.4. Knowledge-Base-Unrelated Diversity

Knowledge-base-unrelated diversity reflects the fraction of a firm's knowledge in unrelated technical fields. Similar to the practices of Chen and Chang (2012) and Krafft et al. (2011), we use information entropy to measure the knowledge-based variety (KBV) and KBUD. The KBV of firm *i* is given by


(5) $$\begin{array}{l} {\text{KBV}}_{i}=\overset{n_{i}}{\underset{j=1}{\sum }}{\text{pro}}_{ij}Ln\left(\frac{1}{{\text{pro}}_{ij}}\right), \end{array}$$

where pro_*ij*_ is the same as given by Equation (2).

The KBUD of firm *i* is


(6) $$\begin{array}{l} {\text{KBUD}}_{i}=\overset{m_{i}}{\underset{k=1}{\sum }}q_{ik}Ln\left(\frac{1}{q_{ik}}\right) \end{array}$$

where *q*_*ik*_ is the proportion of the number of authorized patents of firm *i* in the *k*th technical field to the total number of authorized patents; *m*_*i*_ is the total number of IPC department of authorized patents of firm *i* in all technical fields in the first 3 years.

#### 3.2.5. Knowledge-Base-Related Diversity

The knowledge-base-related diversity (KBRD) reflects the fraction of a firm's knowledge in related technical fields. Based on Equations (5) and (6), the KBRD of firm *i* is


(7) $$\begin{array}{l} {\text{KBRD}}_{i}={\text{KBV}}_{i}-{\text{KBUD}}_{i}. \end{array}$$

Since the collinearity among knowledge-base features is a potential problem, it affects the dual-innovation performance of firms. In order to reduce the interference they may cause, we must eliminate any correlation between KBB, KBD, KBS, KBUD, and KBRD. Upon analyzing their correlation and multicollinearity (Mikalef and Krogstie, 2020), we find that they correlate weakly with each other. In addition, since their variance inflation factors are less than ten, they share no clear multicollinearity. Toward this end, we choose KBB, KBD, KBS, KBUD, and KBRD as the final knowledge-base features of firms. The selection process thus solves the first problem raised in this study.

### 3.3. Measurement of Dual-Innovation Performance

The dual innovation discussed herein includes both exploratory innovation and exploitative innovation. The former mainly focuses on the acquisition and creation of new knowledge and technology, which is an innovative model for firms to seek knowledge and technology from the exterior. The latter is a transformative innovation behavior that need not completely change the original knowledge and technology but only make small-scale changes and innovations. The purpose of exploitative innovation is to improve the current status of a firm and enhance its short-term benefits. To analyze how knowledge-base features affect a firm's dual-innovation performance, we measure the firm's dual-innovation performance as follows.

#### 3.3.1. Exploratory Innovation Performance

This study draws on the method of Gilsing et al. (2008) to distinguish and measure the exploratory innovation performance (EIP1) based on the technology category as represented by the IPC subclass. Taking the technology categories of all patents granted to firms from 2015 to 2017 as the judgment basis, we regard the number of patents granted in the new-patent-technology categories appearing from 2018 to 2020 as a measurement of EIP1.

#### 3.3.2. Exploitative Innovation Performance

Similar to EIP1, this study uses as a basis the technology categories of all patents granted to firms from 2015 to 2017. We take the number of patents granted for common technology categories in the first 3 years (from 2015 to 2017) and in the last 3 years (from 2018 to 2020) to measure exploitative innovation performance (EIP2).

## 4. Research Process

This section constructs a research framework and introduces the corresponding processes used in this study.

### 4.1. Research Framework

**Figure 1:**
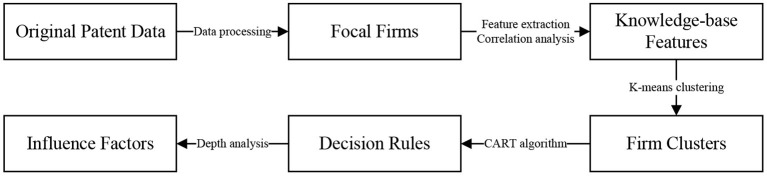
Research framework used in this study.

In this study, we develop a data-driven research framework to analyze the complex nonlinear relationship between knowledge-base features and a firm's dual-innovation performance. As shown in Figure 1, we first obtain some focal firms by processing the original patent data. Next, we select knowledge-base features of focal firms by analyzing the correlation and multicollinearity of variables. Based on the results, we use a k-means clustering algorithm to divide focal firms into different clusters. In addition, we obtain the corresponding decision rules of focal firms in different clusters. Finally, through an in-depth analysis of the decision rules, we provide focal firms with some suggestions. In the simplest terms, this study consists mainly of two important processes: (i) the division of firms and (ii) the acquisition of decision rules. The two processes solve perfectly the last two problems in this study. Concretely, the first process explores which firms contain similar knowledge-base features and which clusters include dissimilar knowledge-base features. The last process reveals the mechanism that connects knowledge-base features and a firm's dual-innovation performance. In other words, we find the detailed factors and decision rules that determine a firm's dual-innovation performance. Next, we will analyze them in detail.

### 4.2. Division of Firms

The previous analysis indicates that knowledge-base features such as KBB, KBD, KBS, KBUD, and KBRD may affect a firm's dual-innovation performance. Given the difference in intrinsic conditions, firms with different knowledge-base features may have different dual-innovation performances and vice versa. To mine a firm's clusters with similar knowledge-base features and further reveal their decision-making rules in a fine-grained manner, we divide firms into different clusters according to their knowledge-base features.

**Figure 2:**
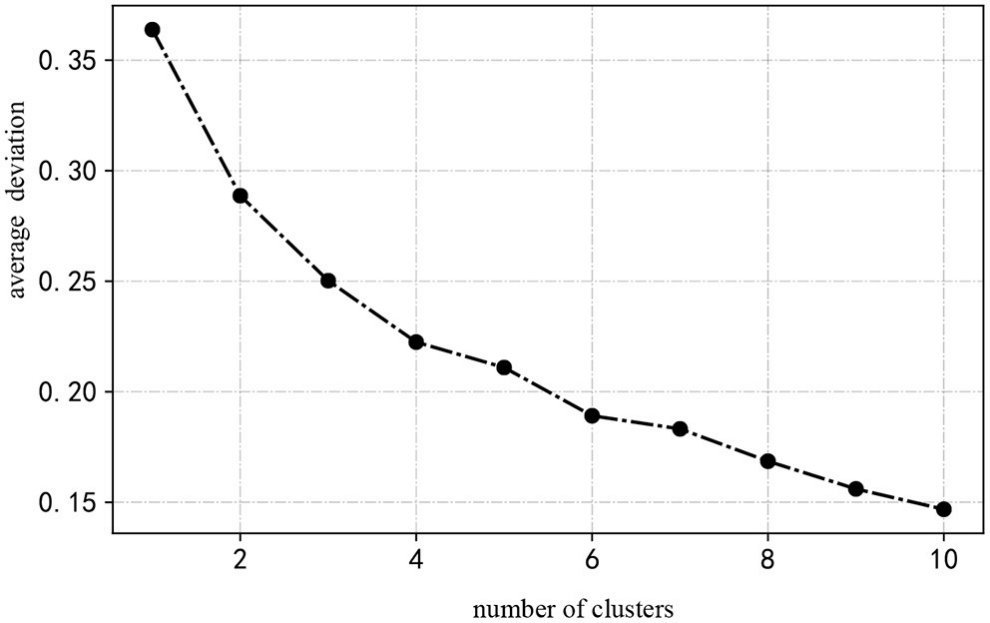
An optimal number of clusters of firms.

Clustering is a popular data mining technique that places records into homogenous groups (Juan Pineda-Jaramillo, 2021). Given that the k-means clustering algorithm is simple in principle and easy to implement, this study uses it to group focal firms to form clusters with similar knowledge-base features. This is done as follows:

1. Make 0–1 standardization for knowledge-base features such as KBB, KBD, KBS, KBUD, and KBRD.
2. Determine the optimal clustering number k by applying the elbow algorithm (Mouton et al., 2020). Its specific operations are as follows: first, we sum the squares of the distances from each point to the center of the cluster to which it belongs. When it slows, it is considered to be the optimal K value. As shown in Figure 2, the number of different firms corresponds to different average dispersions. When the number of clusters is from one to four, the average dispersion in the clustering results varies strongly. Once the number of clusters exceeds four, a small change appears in the average dispersion. Therefore, the optimal number of clusters of firms is *k* = 4.
3. Based on the number of clusters, we use a k-means clustering algorithm to cluster 285 focal firms. Finally, four clusters with similar knowledge-base features are found.

As shown in Table 1, the 285 focal firms can be divided into four different clusters by using the k-means clustering algorithm. The knowledge-base features are the corresponding average value in different clusters. In addition, the numeral “1” in “Proportion of EIP1 and EIP2” means that both EIP1 and EIP2 are high, and zero are low. The levels of EIP1 and EIP2 are determined by the median of the corresponding innovation performance. The innovation performance exceeding the median is set to be high, otherwise the opposite. Firms in different clusters contain some heterogeneity features. The detailed differences are as follows:

1. Eighty-nine focal firms are involved in Cluster I, which accounts for 31.2% of all focal firms. Compared with Cluster III and Cluster IV, the firms in Cluster I have greater KBB and KBRD, which indicates that firm knowledge in Cluster I not only covers a wide range of technical fields but also is distributed more broadly in each technical field. In addition, because KBD, KBS, and KBUD in Cluster I rank third among all clusters, firms in Cluster I have a weak understanding of relevant knowledge fields and, at the same time, their knowledge stock and the knowledge distribution across technical fields are also low. The fraction of high EIP1 and low EIP2 in Cluster I both exceed 50%. Therefore, given the current knowledge base, firms in Cluster I are more inclined to generate high EIP1 and low EIP2.
2. Cluster II has the fewest focal firms, accounting only for 11.9% of all focal firms. Compared with the three other clusters, all knowledge-base features of firms in Cluster II are the largest, which indicates that firms in Cluster II enjoy a rich accumulation of knowledge and are more familiar with knowledge from different technical fields. In addition, the degree of knowledge elements owned by firms in the Cluster II dispersing in different scientific fields and related technical fields is also maximal. Given that the fractions with high EIP1 and high EIP2 both exceed 80%, under the existing knowledge-base level, firms in this cluster not only attach importance to the use of past technologies for R&D but also exceed at excavating and developing new technical fields.
3. The 78 focal firms in Cluster III account for 27.4% of all focal firms. Firms in Cluster III have a higher KBUD; in other words, these firms devote a greater fraction of their knowledge base to irrelevant technical fields than do firms in the other clusters. At this time, more new knowledge elements may be obtained. Furthermore, as shown in Table 1, both KBD and KBS in Cluster III are minimal among all clusters, which implies that firms in Cluster III have insufficient knowledge stock and are extremely unfamiliar with existing knowledge. In addition, given that the fraction of low EIP1 and low EIP2 both exceed 50%, firms in this cluster not only seldom use past technology for R&D and design but also do not excel at mining and developing new technical fields.
4. Cluster IV contains a total of 84 focal firms, which account for 29.5% of all focal firms. As opposed to the other three clusters, firms in this cluster have relatively greater KBD and KBS, which implies that firms in Cluster IV not only are familiar with the relevant knowledge but also have abundant knowledge accumulation. In addition, as shown in Table 1, KBB, KBUD, and KBRD are minimal in Cluster IV, which indicates that the existing knowledge units of firms in this cluster cover the least technical fields, and the fractional distribution of the corresponding knowledge elements in relevant and irrelevant technical fields are also minimal. Furthermore, because the fractions of low EIP1 and high EIP2 in Cluster IV both exceed 50%, under the current knowledge-base level, firms in Cluster IV may focus greater attention on using past technology in R&D and dig less to develop new technical fields.

**Table 1:** Statistical information in different clusters.

**Cluster**	**Number**	**KBB**	**KBD**	**KBS**	**KBUD**	**KBRD**	**Proportion of EIP1**	**Proportion of EIP2**
I	89	5.596	0.775	11.974	0.507	0.828	1: 52.8%	1: 44.9%
							0: 47.2%	0: 55.1%
II	34	23.853	1.536	156.092	0.968	1.323	1: 88.2%	1: 91.2%
							0: 11.8%	0: 8.8%
III	78	4.962	0.695	9.240	0.855	0.404	1: 43.6%	1: 30.8%
							0: 56.4%	0: 69.2%
IV	84	3.464	0.958	34.939	0.177	0.138	1: 45.2%	1: 57.1%
							0: 54.8%	0: 42.9%

In order to determine what types of knowledge-base features are more conducive to improving the dual-innovation performance of firms, we need to further analyze the decision rules of EIP1 and EIP2 in different firm clusters.

### 4.3. Acquisition of Decision Rules

The advantage of the decision tree model is that it captures the interaction between variables and sorts all explanatory variables according to their degree of influence on the dependent variable, thereby organizing better decision management. Therefore, this study uses the CART decision tree algorithm to further analyze the complex nonlinear relationship between knowledge-base features and firms' dual-innovation performance in different clusters. Specifically, we choose KBB, KBD, KBS, KBUD, and KBRD as conditional properties and dual-innovation performance as the decision attribute. Meanwhile, prior to using the CART algorithm to obtain the cluster decision rules, we discretize the firms' dual-innovation performance. The dual-innovation performance exceeding the median of all dual-innovation performance is regarded as high performance; otherwise, it is regarded as low performance. After pruning, we obtain the corresponding decision rules.

## 5. Analysis of Decision Rules

By using the CART algorithm, we obtain the decision rules for EIP1 and EIP2 for four different firm clusters. The following conclusions are drawn from the data given in Table 2: (1) In different firm clusters, two different knowledge-base features may bring about different EIP1 for firms, which demonstrate the necessity of categorizing the different firms. (2) The high EIP1 in Clusters I and II accounts for over 50% of the firms, whereas the other two clusters are just the opposite. These results are entirely consistent with Table 1, which indicates that the CART algorithm used to analyze the decision rules of EIP1 does not modify the distribution of the original EIP1. (3) The degree of confidence of most decision rules exceeds 60%, and some of them exceed 90%, or even 100%, which shows that the decision rules obtained by the CART algorithm has high interpretability.

**Table 2:** Decision rules of exploratory innovation performance (EIP1).

**Cluster**	**KBB**	**KBD**	**KBS**	**KBUD**	**KBRD**	**Support**	**Confidence**	**Result**
I	≤5.5			≤0.563		42.7%	52.6%	High
	≤5.5			>0.563		20.2%	88.9%	Low
	>5.5			≤0.492		10.1%	66.7%	Low
	>5.5			>0.492		27.0%	91.7%	High
II			≤64.167		>1.331	14.7%	60.0%	Low
			>64.167			44.1%	100%	High
			≤64.167		≤1.331	41.2%	93.0%	High
III		≤0.381				17.9%	85.7%	Low
		(0.381,0.699)		≤0.77		15.4%	75.0%	High
		(0.381,0.699)		>0.77		20.5%	68.8%	Low
		(0.699,0.85)				20.5%	87.5%	Low
		>0.85				25.6%	80.0%	High
IV			≤7.8			53.6%	75.6%	Low
		≤0.882	>7.8			9.5%	75.0%	Low
		>0.882	>7.8			36.9%	80.7%	High

Similarly, the results given in Table 3 lead to the following conclusions: (1) At most two knowledge-base features (KBS, KBUD) affect the EIP2 of firms. In particular, EIP2 in Clusters I and III may be affected by KBS and KBUD, whereas EIP2 in Cluster IV is only affected by KBS. Clearly, KBS exists in each decision rule, so it may be a critical factor affecting the EIP2 of firms. (2) The results given in Table 1 show that, although 91.2% of firms in Cluster II have high EIP2, no decision rules exist for EIP2 in this cluster. (3) The degree of confidence of most decision rules exceeds 60%, and over half of the decision rules have a degree of confidence exceeding 90%, which indicates that EIP2, as obtained by the CART algorithm, is credible.

**Table 3:** Decision rules of exploitative innovation performance (EIP2).

**Cluster**	**KBB**	**KBD**	**KBS**	**KBUD**	**KBRD**	**Support**	**Confidence**	**Result**
I			>4.5	>0.44		37.1%	90.9%	High
			>4.5	≤0.44		14.6%	61.5%	Low
			≤4.5			48.3%	88.4%	Low
II								
III			≤4.483			52.6%	92.7%	Low
			(4.483,11.083)	>0.887		14.1%	90.9%	Low
			(4.483,11.083)	≤0.887		14.1%	54.6%	High
			>11.083			19.2%	93.3%	High
IV			≤7.25			50.0%	78.6%	Low
			>7.25			50.0%	92.9%	High

According to the detailed decision rules of firms, we find that the CART algorithm mines multiple knowledge-base features affecting the dual-innovation performance of firms when the sample data do not obey a distribution. This aspect of the CART algorithm not only avoids the limited requirements of traditional regression models regarding data distribution but also reveals the multi-factor nonlinear effects of EIP1 and EIP2. In the next section, we analyze in detail the decision rules in different clusters.

### 5.1. Decision Rules in Cluster I

#### 5.1.1. Decision Rules of EIP1

**Figure 3:**
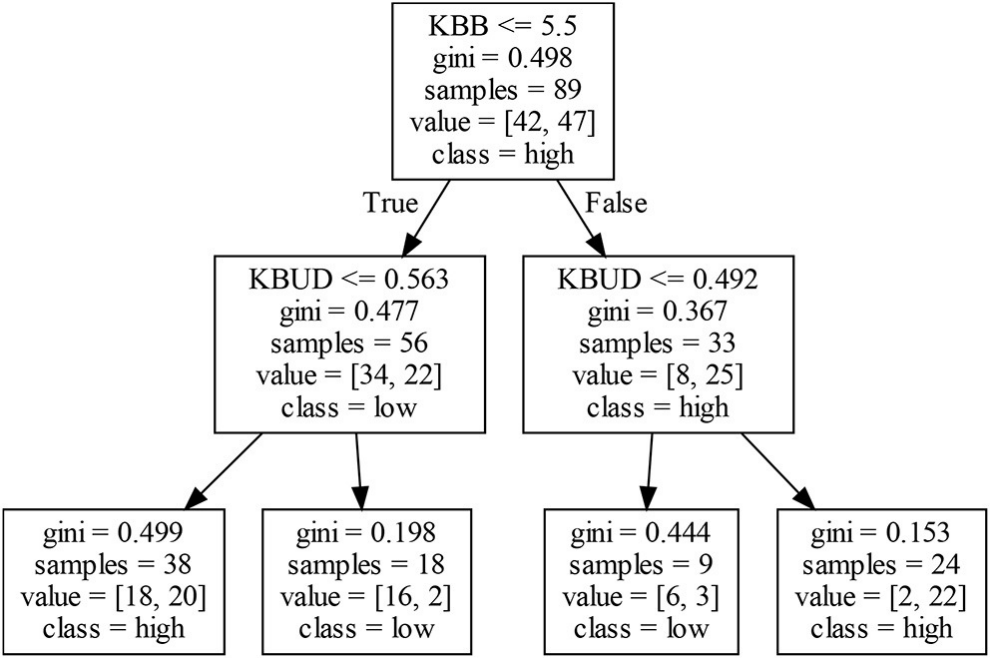
Decision tree for exploratory innovation performance (EIP1) in Cluster I.

Exploratory innovation performance for firms in Cluster I is mainly affected by KBB and KBUD. Analyzing the nodes of the left decision tree in Figure 3 shows that, although both KBB and KBUD are low, a high EIP1 remains possible. That is, although the degree of dispersion of all knowledge units in different scientific fields is not high, firms may still generate high EIP1 due to the low cost of searching and integrating diverse knowledge from different scientific fields. In addition, when knowledge elements owned by firms do not cover a sufficiently wide range of technologies, a higher KBUD can increase the difficulty of integrating knowledge between different scientific fields, which may degrade firms' EIP1. Analyzing the nodes of the right decision tree in Figure 3 shows that, when both KBB and KBUD are high, firms may obtain a high EIP1 because all knowledge owned by firms covers a wide range of technical fields, so the fraction in irrelevant technical fields is also high. At this time, a firm may own more new knowledge elements, which further expands the scope of its knowledge resource and is more conducive to developing its EIP1. To summarize, when KBB is low (high), KBUD may negatively (positively) affect a firm's EIP1.

#### 5.1.2. Decision Rules of EIP2

**Figure 4:**
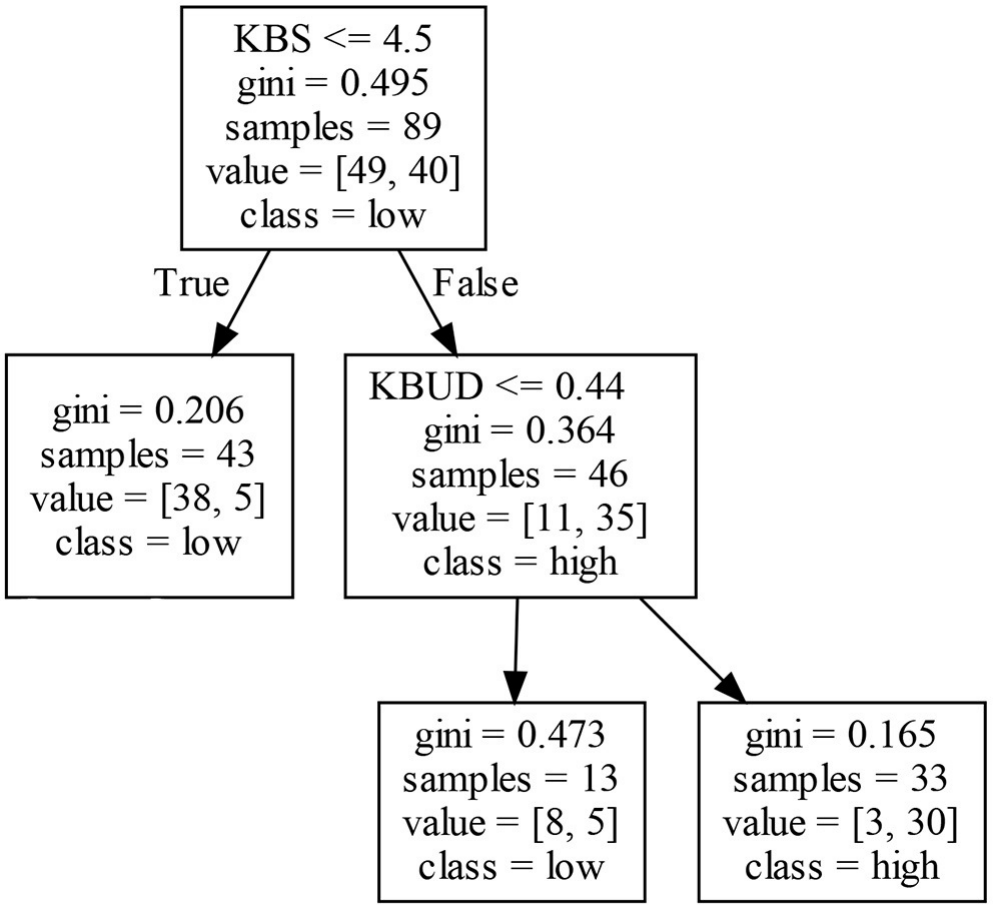
Decision tree for exploitative innovation performance (EIP2) in Cluster I.

As shown in Figure 4, KBS or the combination of KBS and KBUD may affect a firm's EIP2. The left decision tree consists of one KBS node. When the KBS of a firm is low, it is not conducive to innovation, integration, and improvement of the original knowledge and technology by firms because both the knowledge stock and the innovation experience are insufficient. Finally, a firm's EIP2 may be further inhibited. Analyzing the right decision tree in Figure 4 shows that firms with a higher KBS may not obtain high EIP2. If a firm's KBUD is also high, then the firm may obtain a high EIP2; otherwise, the EIP2 will be low. This seems to indicate that high knowledge accumulation and a high knowledge fraction in irrelevant technical fields lead to a high EIP2. To summarize, KBS is not the only feature determining a firm's EIP2—sometimes it needs to be adjusted in conjunction with KBUD.

### 5.2. Decision Rules in Cluster II

#### 5.2.1. Decision Rules of EIP1

**Figure 5:**
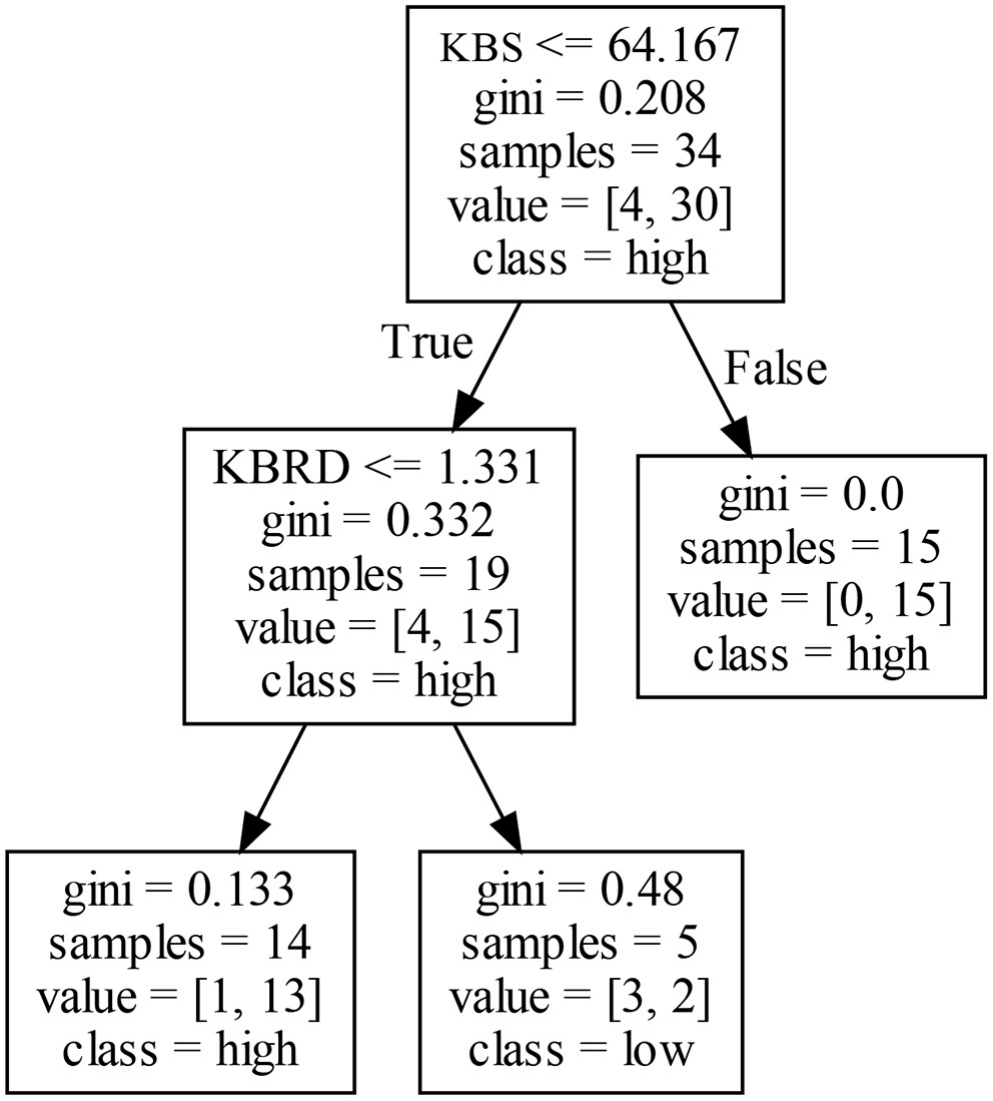
Decision tree for EIP1 in Cluster II.

A firm's EIP1 in Cluster II is mainly affected by KBS and KBRD. Different combinations of KBS and KBRD may produce different results for EIP1. Analyzing the left decision tree in Figure 5 shows that both KBS and KBRD affect a firm's EIP1. When KBS is low, KBRD may negatively affect a firm's EIP1. In other words, if the knowledge stock of a firm is deficient and if the fraction of technology resources allocated by firms in the same technology field is low, firms may generate a high EIP1 by reducing the homogenization of knowledge and strengthening the absorption and use of diversified knowledge. Analyzing the right decision tree in Figure 5 shows that only KBS affects a firm's EIP1. Firms with a higher knowledge accumulation may produce more innovation achievements and accumulate more innovation experience, which helps firms increase their EIP1. A horizontal comparative analysis shows that a high KBS may lead to a high EIP1, whereas a low KBS does not completely determine a firm's EIP1. If a firm has a lower knowledge accumulation, KBRD may negatively affect its EIP1.

#### 5.2.2. Decision Rules of EIP2

There are no decision rules for EIP2 exist in Cluster II. However, this does not mean that the knowledge-base features in this cluster do not affect the firms' EIP2, but rather that no detailed rules affect the EIP2 of firms in this cluster. To investigate this phenomenon in detail, we analyze it from the perspective of statistics. As shown in Table 4, the fluctuations of knowledge-base features and EIP2 in this cluster differ significantly. The fluctuations of KBB, KBD, KBUD, and KBRD are relatively gentle, whereas KBS and EIP2 fluctuate strongly because the standard deviations (Std.) of KBS and EIP2 exceed the corresponding averages. In addition, as shown in Table 1, all the knowledge-base features of firms in this cluster are maximal, and the corresponding EIP2 is also as high as 91.2%. On the one hand, this confirms that a strong knowledge-base leads to high EIP2. On the other hand, it also reflects the reliability of firm clustering based on knowledge-base features. To summarize, the complex fluctuations and the maximum strength of knowledge-base features in this cluster make it difficult for firms in this cluster to find the detailed decision rules of EIP2 by using the CART algorithm.

**Table 4:** Descriptive statistics of variables.

**Variables**	***N***	**Min**.	**Max**.	**Mean**	**Std**.
KBB	34	10.000	75.000	23.850	15.096
KBD	34	0.311	3.601	1.536	0.712
KBS	34	5.667	1266.700	156.092	256.821
KBUD	34	0.597	1.270	0.968	0.145
KBRD	34	0.893	1.896	1.323	0.253
EIP2	34	2.000	1265.100	147.200	240.918

### 5.3. Decision Rules in Cluster III

#### 5.3.1. Decision Rules of EIP1

**Figure 6:**
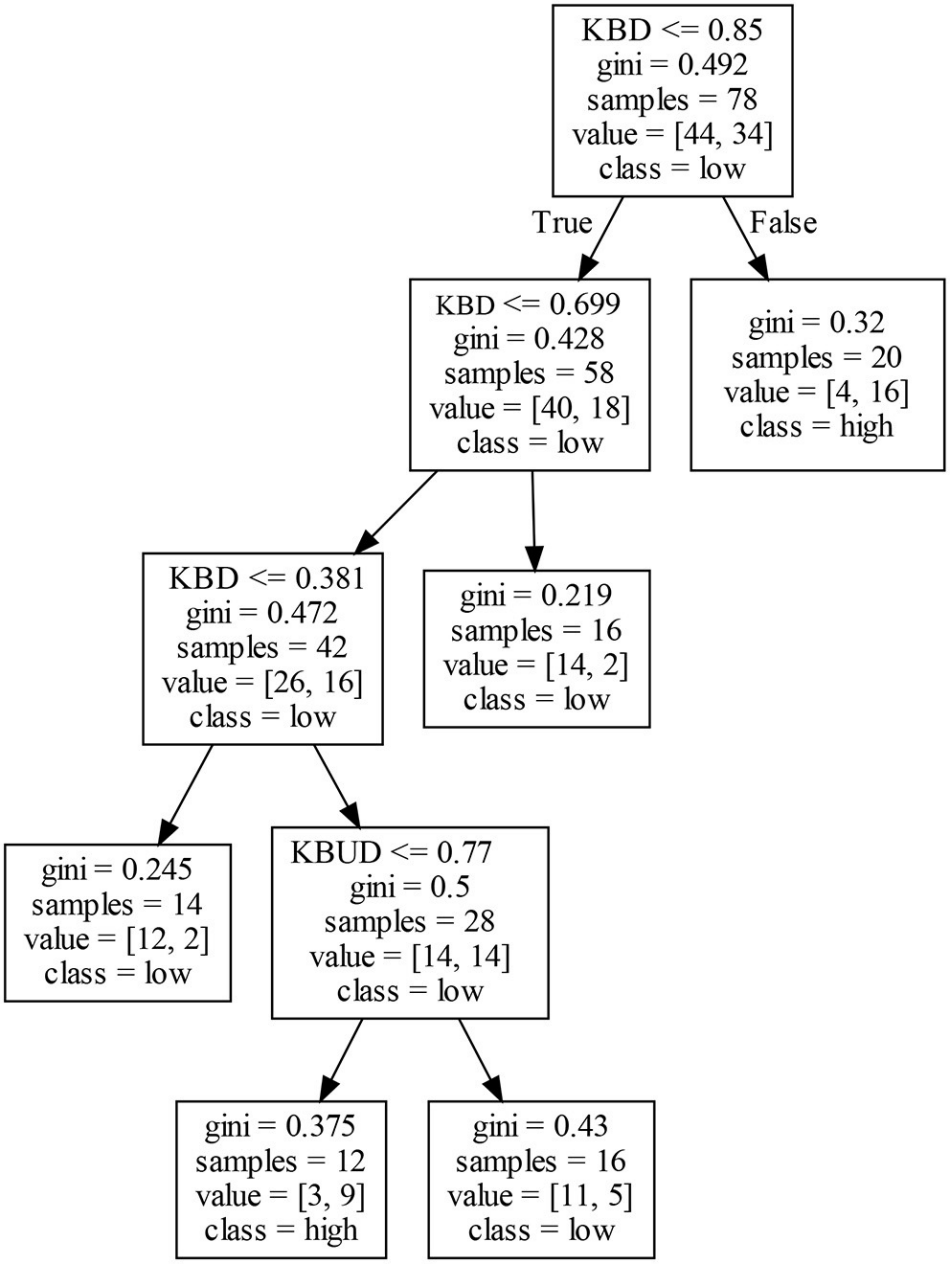
Decision tree for EIP1 in Cluster III.

The exploratory innovation performance of firms in Cluster III may be affected by KBD and KBUD. The right decision tree in Figure 6 shows that firms with a higher KBD may obtain high EIP1. They may be more familiar with existing knowledge and technologies and be able to solve frontier and complex problems, which reduces the cost of communicating information between firms. Meanwhile, the relevant technical opportunities are also detected in time, while it remains possible to improve a firm's EIP1. In addition, an analysis of the left decision tree in Figure 6 shows that different combinations of KBS and KBUD may produce different results for a firm's EIP1. In general, a lower KBD correlates with a lower EIP1. Firms with a moderate KBD may have a low EIP1 or may need to use KBUD to negatively adjust their EIP1.

#### 5.3.2. Decision Rules of EIP2

**Figure 7:**
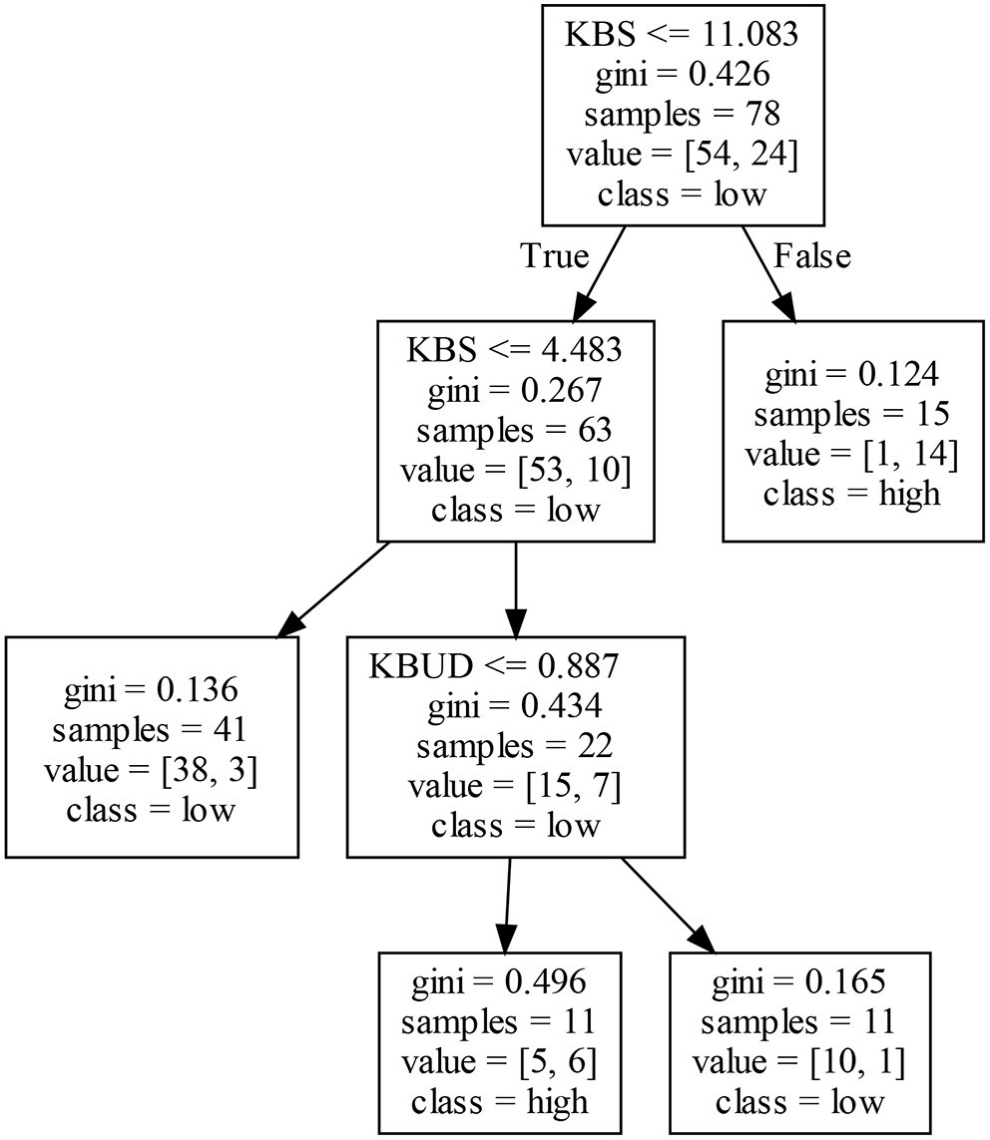
Decision tree for EIP2 in Cluster III.

Figure 7 shows that, in Cluster III, a firm's EIP2 is mainly affected by KBS and KBUD, and different combinations of the two may produce different results for EIP2. In general, firms with a higher (lower) KBS may achieve a higher (lower) EIP2. In particular, firms with a moderate level of KBS obtain different EIP2 by adjusting their level of KBUD. The right decision tree in Figure 7 shows that firms with abundant knowledge accumulation generate more innovation results and accumulate more innovation experience, which promotes innovation activities. An analysis of the left decision tree in Figure 7 shows that firms with a lower knowledge stock have insufficient innovation experience, which does not help to enhance a firm's EIP2. In addition, when a firm's KBS is at the medium level, KBUD may negatively affect its EIP2. In other words, when firms have a certain knowledge stock, a higher KBUD may make it harder to integrate knowledge between scientific fields, which may inhibit a firm's EIP2. However, if KBUD is low due to the ability to integrate existing knowledge, a firm may generate a high EIP2.

### 5.4. Decision Rules in Cluster IV

#### 5.4.1. Decision Rules of EIP1

**Figure 8:**
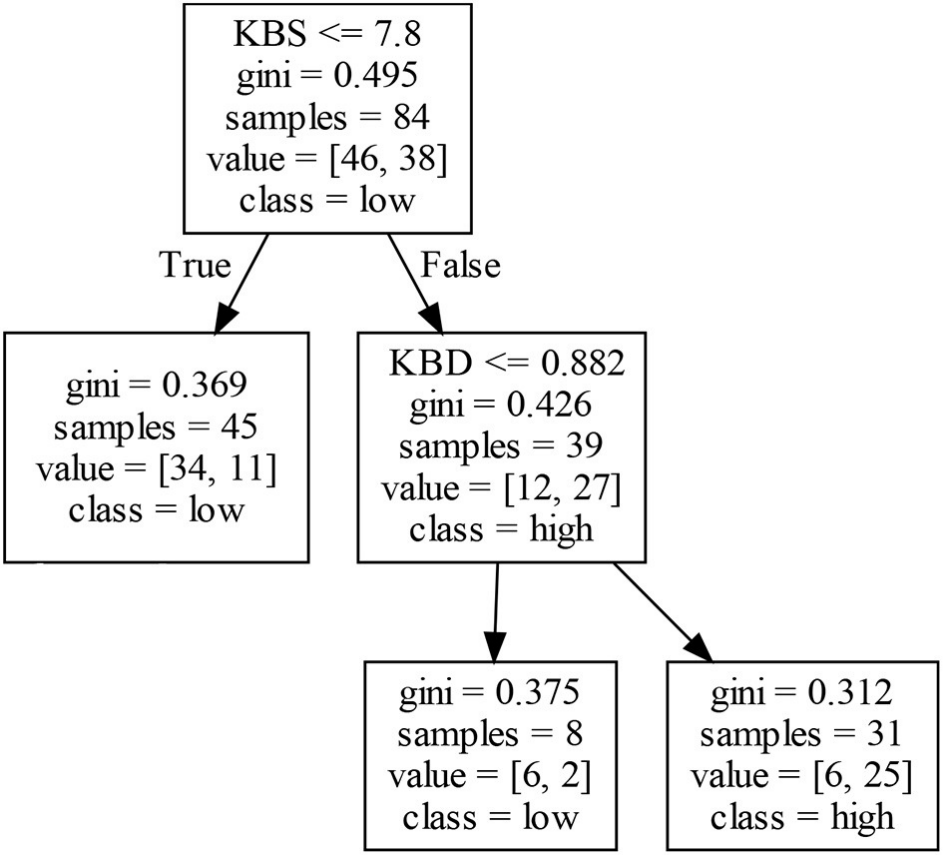
Decision tree for EIP1 in Cluster IV.

As shown in Figure 8, a firm's EIP1 in Cluster IV is mainly affected by KBS and KBD. The left decision tree in Figure 8 shows that firms with a lower KBS may obtain low EIP1. That is, when a firm has insufficient knowledge stock, its EIP1 will be restrained to a certain extent because the innovation experience accumulated by the firm remains deficient. In addition, an analysis of the right decision tree in Figure 8 shows that KBS does not completely determine a firm's EIP1, and different combinations of KBS and KBD may produce different EIP1. For example, firms with a higher KBS and a lower KBD may produce a low EIP1. In other words, although firms have sufficient knowledge stock, the insufficient understanding of firms in the relevant knowledge fields facilitates inconsistency when firms exchange information with each other. This clearly increases the communication cost of firms and reduces the opportunities to identify related technologies, which hinders the generation of EIP1. To summarize, if KBS is low, the related firms may produce a low EIP1. However, if KBS is high, KBD may positively affect a firm's EIP1.

#### 5.4.2. Decision Rules of EIP2

**Figure 9:**
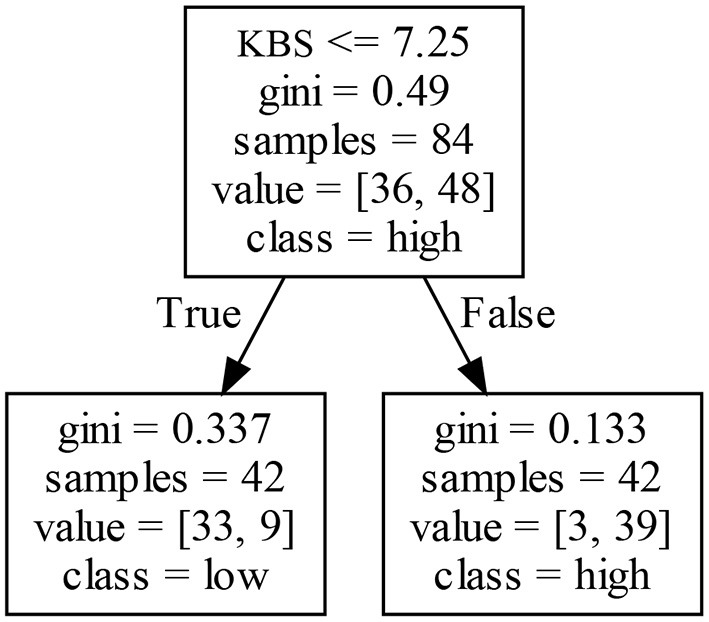
Decision tree for EIP2 in Cluster IV.

Figure 9 shows that KBS positively affects a firm's EIP2 in Cluster IV. That is, firms with a higher (lower) KBS obtains a high (low) EIP2. This result may be because firms with different accumulations of existing knowledge may obtain different technological innovation achievements and innovation experiences.

## 6. Conclusion and Discussion

### 6.1. Conclusion

This study takes 285 focal firms from the field of Chinese new-energy vehicles as the research object and uses the k-means clustering algorithm to cluster firms with similar knowledge-base features. For firms in different clusters, we use KBB, KBD, KBS, KBUD, and KBRD as conditional attributes and the discretized dual-innovation performance as the decision attribute. By using the CART algorithm, we discover a series of decision rules that affect a firm's dual-innovation performance. In particular, we obtain the following results:

1. Four different firm clusters are obtained with clear differences between knowledge-base features in the different clusters. The influence of knowledge-base features on EIP1 and EIP2 is different. Five features of knowledge-base breadth, depth, scale, unrelated and related diversity all jointly affect the EIP1 in the form of pairwise combinations. Meanwhile, the combination of knowledge-base features affecting EIP1 in different clusters of firms is also different. In addition, for the EIP2, only both KBS and KBUD have an impact on it.
2. The EIP1 of firms in Cluster I is mainly affected by KBB and KBUD. In particular, if firms have a lower KBB, KBUD will negatively affect their EIP1. Otherwise, KBUD will positively affect the EIP1 of firms with a higher KBB. In addition, the EIP2 of firms in Cluster I is mainly impacted by KBS and KBUD. Firms with a lower KBS may obtain low EIP2. However, firms with a higher KBS find their EIP2 to be positively regulated through KBUD.
3. The knowledge-base features of firms in Cluster II are the largest so that the fraction of firms having both high EIP1 and high EIP2 exceeds 85%. Meanwhile, the EIP1 of firms in this cluster is mainly affected by KBS and KBRD. In particular, firms with a higher KBS may obtain a high EIP1. However, for firms with a lower KBS, KBRD may negatively affect their EIP1. In addition, no decision rules exist in Cluster II for a firm's EIP2.
4. The EIP1 of firms in Cluster III is mainly affected by KBD and KBUD. In particular, firms with a lower (higher) KBD have a low (high) EIP1. However, if a firm has a moderate KBD, it may obtain a low EIP1 or need to use KBUD to negatively adjust its EIP1. In addition, EIP2 of firms in Cluster II is mainly affected by KBS and KBUD. Firms with a lower (higher) KBS will have a low (high) EIP2. Firms with an appropriate level of KBS have EIP2 negatively regulated by KBUD.
5. The EIP1 of firms in Cluster IV is mainly affected by KBD and KBS. In particular, if firms have a lower KBS, they may obtain a low EIP1. Firms with a higher KBS find that KBD may positively affect their EIP1. In addition, the EIP2 of firms in Cluster IV is positively affected by KBS.

### 6.2. Management Implications

Based on the results in the different clusters, we make the following suggestions.

1. For some firms with a higher KBB and KBRD and a lower KBD, KBS, and KBUD, if their knowledge units cover fewer technical fields, they should reduce the fraction of technical resources allocated to different technical fields to increase their EIP1. Otherwise, they should increase the fraction. In addition, to increase their EIP2, these firms not only need to accumulate sufficient technology and knowledge as early as possible but also need to increase the investment ratio of technical resources in different technical fields.
2. Firms with the maximum KBB, KBD, KBS, KBUD, and KBRD can increase their EIP1 by raising their level of knowledge stock. If their knowledge accumulation is insufficient, they should consider reducing the fraction of technical resources in the same technical field.
3. For firms with the minimum KBD and KBS, if their KBB and KBRD are also low, they should strengthen their understanding of relevant knowledge areas to increase their EIP1. If they are still not familiar with the technology and knowledge of the industry, they should reduce the allocation ratio of technical resources in different technical fields. In addition, if these firms want to obtain a high level of EIP2, they should increase their knowledge stock as much as possible by strengthening exchange and cooperation with external organizations. Conversely, if their knowledge accumulation is insufficient, they should reduce the fraction of technical resources allocated to different technical fields.
4. For firms that have higher KBD and KBS, if their KBB, KBUD, and KBRD are minimal, they should increase their own knowledge stock and understanding by strengthening the scope and depth of their technical and knowledge exchanges with external organizations. In addition, to increase their EIP2, they should strengthen their exchange and cooperation with external organizations to accumulate more innovation achievements and experiences.

### 6.3. Theoretical Contributions

This study not only provides firms containing different knowledge base with the development suggestion, but also produces the following theoretical contributions.

1. This study promotes the integration and development of some theories such as knowledge base, exploratory innovation, exploitative innovation, and data mining. Knowledge base are regarded as an important source of firms' sustainable competitive advantage (Yu and Yan, 2021; Low and Ho, 2016). Based on the knowledge-based view and data mining and other related theories, this study constructs the theoretical model of the relationship between the knowledge base and dual-innovation performance of firms and analyzes the influence of knowledge-base features on the dual-innovation performance of firms in different clusters. The multi-factor combination effect of dual-innovation performance of firms is further identified. To a certain degree, the relevant conclusions advance the integration and development of knowledge-based view, exploratory innovation theory, exploitative innovation theory, and data mining theory. Meanwhile, they also lay a foundation for the research on the influence mechanism between variables in the future.
2. The multi-factor influence mechanism of a firm's dual-innovation performance is objectively determined. In reality, many factors affect a firm's dual-innovation performance, but most factors obtained by previous empirical research are linear or simple nonlinear factors. Any correlation or inherent complex nonlinear relationships between factors are often ignored. In addition, traditional methods often have trouble understanding multi-factor interactions. The present study uses the k-means and CART algorithms from the field of machine learning to analyze the factors that affect a firm's dual-innovation performance, which not only compensates for the inability of traditional regression methods to analyze how different combined characteristic factors affect the explained variables but also reveals the multi-factor effect of knowledge-base features on a firm's dual-innovation performance.
3. The quality of the analysis of factors affecting the dual-innovation performance of firms is improved. The current research often constructs linear or simple nonlinear hypotheses of single or multiple independent variables to the dependent variable based on literature organization and then verifies them through a questionnaire survey. Given the interference of external factors in the process of scale design, measurement, data collection, and the limitation of sample size, some results may be subjective and unstable. Although a small number of studies analyze the dual-innovation performance of firms by using objective second-hand patent data, the complex nonlinear relationship between independent variables and dependent variables is often ignored. This study divides firms with similar knowledge-base features into the same cluster and mainly analyzes the influence of knowledge-base features on the dual-innovation performance of firms in different clusters, making the research results more targeted. Meanwhile, the application of second-hand patent data and data-driven method also further improves the reliability of results.

### 6.4. Limitations and Future Research

The first limitation of this study is that certain outliers may exist in the original patent data, so valuable information hidden in them should be further mined. However, most of the time, these outliers may need to be cleaned with the help of the isolation forest algorithm (Tokovarov and Karczmarek, 2022). Future studies may include the analysis of outliers in the sample data. Second, the selected knowledge-base features may not fully represent a firm's knowledge base. For example, knowledge-base consistency (Saviotti, 2005) and some relational characteristics between knowledge elements, such as knowledge substitutability, complementarity, and variety (Xu and Zeng, 2021), may also affect a firm's innovation performance. Future studies may consider their influence on a firm's dual-innovation performance. Finally, the division of firms by the k-means algorithm requires *a priori* knowledge of the number of clusters. Future studies may introduce the adaptive affinity propagation clustering algorithm to accurately divide firms.

## Data Availability Statement

The original contributions presented in the study are included in the article/supplementary material, further inquiries can be directed to the corresponding author.

## Author Contributions

LZ: data curation, validation, methodology, and writing—original draft. HL: conceptualization, validation, and supervision. CL: conceptualization and validation. XW: writing—review and editing and supervision. All authors contributed to the article and approved the submitted version.

## Funding

This work was supported by Huaqiao University's Academic Project Supported by the Fundamental Research Funds for the Central Universities (21SKGC-QT06).

## Conflict of Interest

The authors declare that the research was conducted in the absence of any commercial or financial relationships that could be construed as a potential conflict of interest.

## Publisher's Note

All claims expressed in this article are solely those of the authors and do not necessarily represent those of their affiliated organizations, or those of the publisher, the editors and the reviewers. Any product that may be evaluated in this article, or claim that may be made by its manufacturer, is not guaranteed or endorsed by the publisher.
